# Sequence Heterogeneity in NS5A of Hepatitis C Virus Genotypes 2a and 2b and Clinical Outcome of Pegylated-Interferon/Ribavirin Therapy

**DOI:** 10.1371/journal.pone.0030513

**Published:** 2012-02-02

**Authors:** Ahmed El-Shamy, Ikuo Shoji, Soo-Ryang Kim, Yoshihiro Ide, Susumu Imoto, Lin Deng, Seitetsu Yoon, Takashi Fujisawa, Satoshi Tani, Yoshihiko Yano, Yasushi Seo, Takeshi Azuma, Hak Hotta

**Affiliations:** 1 Division of Microbiology, Center for Infectious Diseases, Kobe University Graduate School of Medicine, Kobe, Japan; 2 Department of Virology, Suez Canal University Faculty of Veterinary Medicine, Ismalia, Egypt; 3 Division of Gastroenterology, Kobe Asahi Hospital, Kobe, Japan; 4 Department of Gastroenterology, Hyogo Prefectural Kakogawa Medical Center, Kakogawa, Hyogo, Japan; 5 Department of Internal Medicine, Nippon Steel Hirohata Hospital, Himeji, Hyogo, Japan; 6 Department of Internal Medicine, Konan Hospital, Kobe, Japan; 7 Division of Gastroenterology, Department of Internal Medicine, Kobe University Graduate School of Medicine, Kobe, Japan; Saint Louis University, United States of America

## Abstract

Pegylated-interferon plus ribavirin (PEG-IFN/RBV) therapy is a current standard treatment for chronic hepatitis C. We previously reported that the viral sequence heterogeneity of part of NS5A, referred to as the IFN/RBV resistance-determining region (IRRDR), and a mutation at position 70 of the core protein of hepatitis C virus genotype 1b (HCV-1b) are significantly correlated with the outcome of PEG-IFN/RBV treatment. Here, we aimed to investigate the impact of viral genetic variations within the NS5A and core regions of other genotypes, HCV-2a and HCV-2b, on PEG-IFN/RBV treatment outcome. Pretreatment sequences of NS5A and core regions were analyzed in 112 patients infected with HCV-2a or HCV-2b, who were treated with PEG-IFN/RBV for 24 weeks and followed up for another 24 weeks. The results demonstrated that HCV-2a isolates with 4 or more mutations in IRRDR (IRRDR[2a]≥4) was significantly associated with rapid virological response at week 4 (RVR) and sustained virological response (SVR). Also, another region of NS5A that corresponds to part of the IFN sensitivity-determining region (ISDR) plus its carboxy-flanking region, which we referred to as ISDR/+C[2a], was significantly associated with SVR in patients infected with HCV-2a. Multivariate analysis revealed that IRRDR[2a]≥4 was the only independent predictive factor for SVR. As for HCV-2b infection, an N-terminal half of IRRDR having two or more mutations (IRRDR[2b]/N≥2) was significantly associated with RVR, but not with SVR. No significant correlation was observed between core protein polymorphism and PEG-IFN/RBV treatment outcome in HCV-2a or HCV-2b infection. ***Conclusion:*** The present results suggest that sequence heterogeneity of NS5A of HCV-2a (IRRDR[2a]≥4 and ISDR/+C[2a]), and that of HCV-2b (IRRDR[2b]/N≥2) to a lesser extent, is involved in determining the viral sensitivity to PEG-IFN/RBV therapy.

## Introduction

Hepatitis C virus (HCV) is a major cause of chronic liver disease, such as chronic hepatitis, liver cirrhosis and hepatocellular carcinoma, with180 million people being currently infected with HCV worldwide. It is estimated that 70% of acute infections become persistent [1]. As a consequence of the long-term persistence of HCV infection, the number of patients with hepatocellular carcinoma is expected to increase further over the next 20 years. More than two decades have passed since the discovery of HCV, and yet therapeutic options remain limited. Standard regimens for treatment of chronic hepatitis C include pegylated interferon alpha (PEG-IFN) and ribavirin (RBV) [2]. In addition, two protease inhibitors (telaprevir and boceprevir) were approved in May 2011 by the U. S. Food and Drug Administration (FDA) for clinical use in combination with PEG-IFN/RBV to treat chronic hepatitis C patients with HCV genotype 1 [3], [4].

In Japan, about 70% of HCV-infected patients are infected with HCV genotype 1b (HCV-1b) and most of the remaining patients are infected with HCV-2a (25%) or HCV-2b (5%) [5]. When treated with PEG-IFN/RBV, the sustained virological response (SVR) rate is ca. 50% in HCV-1b infection, and ca. 80% in HCV-2a and -2b infections [2], [6]. The mechanism(s) underlying the different responses among patients with different HCV genotypes and subtypes is still unclear. However, this suggests that viral genetic heterogeneity could affect, at least to some extent, the sensitivity to IFN-based therapy. In this context, sequence heterogeneity of the viral NS5A protein has been widely discussed for its correlation with IFN responsiveness. Sequence variations within a region in NS5A of HCV-1b defined as the IFN sensitivity-determining region (ISDR) is correlated with IFN responsiveness [7]. In HCV-2a infection, the influence of sequence heterogeneity in and around a region corresponding to ISDR on the IFN responsiveness was also suggested [8]–[10]. Recently, we identified a new region near the C-terminus of NS5A of HCV-1b, which we refer to as the IFN/RBV resistance-determining region (IRRDR) [11], [12]. The degree of sequence variation within IRRDR was significantly correlated with the clinical outcome of PEG-IFN/RBV combination therapy. The significance of IRRDR of other HCV genotypes, however, has not been investigated yet.

In addition to the NS5A sequence variation, HCV core protein polymorphism was also proposed as a pretreatment predictor of poor virological response in HCV-1b-infected patients treated with PEG-IFN/RBV therapy [13]. It is not clear at this stage whether core protein polymorphism could be used to predict the treatment outcome in HCV-2a and -2b infections. In the present study, we investigated the impact of viral genetic heterogeneity in the NS5A and core regions of HCV-2a and -2b on PEG-IFN/RBV treatment outcome. To the best of our knowledge, this is the first report describing the possible correlation between PEG-IFN/RBV responsiveness and NS5A-IRRDR heterogeneity of HCV-2a and -2b.

## Materials and Methods

### Ethics statement

The study protocol, which conforms to the provisions of the Declaration of Helsinki, was approved beforehand by the Ethic Committees in Kobe Asahi Hospital and Kobe University, and written informed consent was obtained from each patient prior to the treatment.

### Patients

A total of 112 patients seen at Kobe Asahi Hospital and Kobe University Hospital, Kobe, Japan, who were chronically infected with HCV-2a (61 patients) or HCV-2b (51 patients), were enrolled in the study. HCV subtype was determined according to the method of Okamoto et al. [14]. The patients were treated with PEG-IFN α-2b (Pegintron®; Schering-Plough, Kenilworth, NJ) (1.5 µg per kilogram body weight, once weekly, subcutaneously) and RBV (Rebetol®; Schering-Plough) (600∼800 mg daily, per os), for 24 weeks according to a standard treatment protocol for Japanese patients established by a hepatitis study group of the Ministry of Health, Labour and Welfare, Japan. All patients received >80% of scheduled dosage of PEG-IFN and RBV. Serum samples were collected from the patients at intervals of 4 weeks before, during and after the treatment, and tested for HCV RNA and core antigen titers as reported previously [15].

### Sequence analysis of the NS5A and core regions

HCV RNA was extracted from 140 µl of serum using a commercially available kit (QIAmp viral RNA kit; QIAGEN, Tokyo, Japan). The extracted RNA was reverse transcribed and amplified for NS5A and core regions using Super script III one step RT-PCR platinum Taq HiFi (Invitrogen, Tokyo, Japan). The resultant RT-PCR product was subjected to a second-round PCR by using Platinum Taq DNA polymerase high fidelity III (Invitrogen). Primers used for amplification of full-length NS5A of the HCV-2a and -2b genomes and those of the core region of HCV-2a were reported previously [16], [17]. Primers for amplification of the core region of HCV-2b are as follows: C-2b/1 (5′-AGCCATAGTGGTCTGCGGAACC-3′; sense, nucleotides [nt] 136 to 157) and C-2b/4 (5′-GGAACARTTGCACTCTTGGGTG-3′; antisense, nt 1241 to 1262) for one step RT-PCR; C-2b/2 (5′-CCACTCTATGTCCGGTCATTTGG-3′; sense, nt 208 to 230) and C-2b/3 (5′-GAGCTGCCAGGTGATGCTG-3′; antisense, nt 971 to 989) for the second round PCR. RT was performed at 45°C for 30 min and terminated at 94°C for 2 min, followed by the first-round PCR over 35 cycles, with each cycle consisting of denaturation at 94°C for 30 sec, annealing at 55°C for 30 sec and extension at 68°C for 90 sec. The second-round PCR was performed under the same condition. The sequences of the amplified fragments were determined by direct sequencing without subcloning. The amino acid (aa) sequences were deduced and aligned using GENETYX Win software version 7.0 (GENETYX Corp., Tokyo, Japan). The numbering of aa residues for HCV-2a and -2b isolates is according to the polyprotein of HCV-J6 [18] and -J8 [19], respectively.

### Statistical analysis

Numerical data were analyzed by *S*tudent's *t* test while categorical data by Fisher's exact probability test [8]. To evaluate the optimal threshold of the number of aa mutations in ISDR and IRRDR for prediction of treatment outcomes, the receiver operating characteristic curve was constructed. Univariate and multivariate logistic regression analyses were performed to identify independent predictors for treatment outcomes. All statistical analyses were performed using the SPSS version 16 software (SPSS Inc., Chicago, IL). Unless otherwise stated, a *P* value of <0.05 was considered statistically significant.

### Nucleotide sequence accession numbers

The sequence data reported in this paper have been deposited in the DDBJ/EMBL/GenBank nucleotide sequence databases with the accession numbers AB600751 through AB600834.

## Results

### Patients' Responses to PEG-IFN/RBV Combination Therapy in HCV-2a and HCV-2b infections

Of the 61 patients infected with HCV-2a, 46 (75%) patients cleared HCV viremia by week 4 (rapid virological response [RVR]), and all the patients (100%) by week 12 (early virological response [EVR]) and at week 24 (end-of-treatment response [ETR]) (Table 1). Likewise, of 51 patients infected with HCV-2b, 34 (67%), 51 (100%) and 50 (98%) patients achieved RVR, EVR and ETR, respectively. After the end of treatment, 105 patients (58 with HCV-2a and 47 with HCV-2b) could be followed up for another 24 weeks. At the end, SVR was achieved by 49 (84%) patients infected with HCV-2a and by 34 (72%) patients with HCV-2b. Only 9 (16%) and 13 (28%) patients with HCV-2a and -2b, respectively, were non-SVR. There was no case of null-response (continuous viremia throughout the treatment and follow up periods) since all the non-SVR patients once cleared viremia at a certain time point followed by a rebound in viremia either before or after the end of the treatment (relapse).

**Table 1:** Proportions of various virological responses of HCV-2a- and HCV-2b-infected patients treated with PEG-IFN/RBV.

Response	Proportion		
	HCV-2a	HCV-2b	All
RVR	46/61* (75%)	34/51 (67%)	80/112 (71%)
Non-RVR	15/61 (25%)	17/51 (33%)	32/112 (29%)
EVR	61/61 (100%)	51/51 (100%)	112/112 (100%)
ETR	61/61 (100%)	50/51 (98%)	111/112 (99%)
SVR	49/58 (84%)	34/47 (72%)	83/105 (79%)
Non-SVR	9/58 (16%)	13/47 (28%)	22/105 (21%)

*No. of patients/no. of total.

Abbreviations: RVR, rapid virological response; EVR, early virological response; ETR, end-of-treatment response; SVR, sustained virological response.

Comparison of the base line demographic characteristics between SVR and non-SVR patients revealed that, in HCV-2a infection, SVR patients had a significantly lower average age than that of non-SVR (Table 2). In HCV-2b infection, on the other hand, SVR patients had significantly γ-GTP levels than those of non-SVR. There was no significant difference in viremia titers between SVR and non-SVR in patients infected with HCV-2a or -2b.

**Table 2:** Demographic characteristics of HCV-2a- and HCV-2b-infected patients with SVR and non-SVR.

Factor	HCV-2a			HCV-2b		
	SVR	Non-SVR	*P* value	SVR	Non-SVR	*P* value
Age	49.78±13.67*	62.89±7.01	**0.007**	50.03±15.03	55.08±11.22	0.28
Sex (male/female)	22/27	3/6	0.72	17/17	8/5	0.53
Body weight (kg)	60.39±11.00	54.67±10.51	0.15	57.72±13.46	65.08±7.26	0.06
Platelets (×10^4^/mm^3^)	18.54±5.71	19.43±10.78	0.72	17.57±5.65	15.20±7.281	0.27
Hemoglobin (g/dl)	14.38±6.07	14.0±1.56	0.88	14.19±1.59	13.78±1.5	0.49
γ-GTP (IU/L)	37.66±53.25	36.83±24.82	0.97	39.68±34.33	81.30±69.11	**0.02**
ALT (IU/L)	64.75±52.45	94.38±141.3	0.28	86.35±91.95	86.85±118.7	0.98
HCV-RNA (KIU/ml)	1350±1424	1598±1464	0.63	5543±7643	7905±14210	0.47
HCV core antigen (fmol/L)	6543±6927	6105±8290	0.91	9054±6743	9390±8723	0.92

*Mean ± S.D.

Abbreviations: SVR, sustained virological response; γ-GTP, gamma glutamyl transpeptidase; ALT, alanine aminotransferase.

### Sequence Analysis of NS5A of HCV-2a and HCV-2b

The entire NS5A region of the HCV-2a and -2b genomes in pretreatment sera were sequenced, and aa sequences deduced. All the sequences obtained were aligned and the consensus sequences for HCV-2a and -2b were inferred. An N-terminal half (aa 1977 to 2196) of the consensus sequences of HCV-2a and -2b isolates were each identical to the prototype sequences, HCV-J6 [18] and HCV-J8 [19], respectively. The remaining C-terminal half (aa 2197 to 2442) of the consensus sequences were identical to those reported by Murakami et al. [8] except that His at position 2358 in the HCV-2b sequence was replaced with Cys, which was more conserved (59% of the isolates tested) than His (22%).

**Figure 1:**
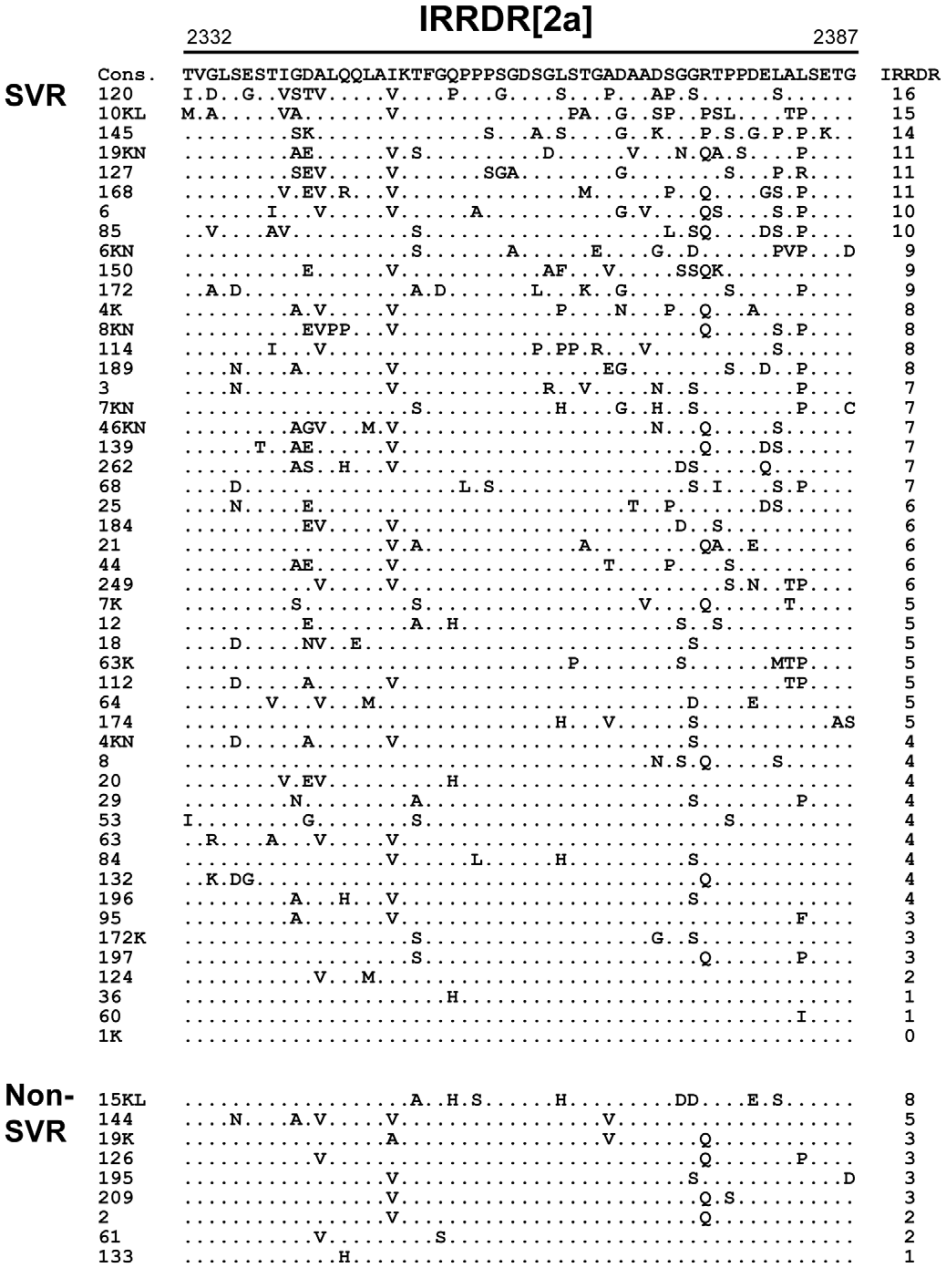
Sequence alignment of IRRDR[2a]. Sequences of IRRDR[2a] (interferon/ribavirin resistance-determining region of HCV-2a) obtained from SVR and non-SVR patients are aligned. The consensus sequence (Cons) is shown on the top. The numbers along the sequence indicate the aa positions. Dots indicate residues identical to those of the Cons sequence. The numbers of the mutations in IRRDR[2a] are shown on the right.

**Figure 2:**
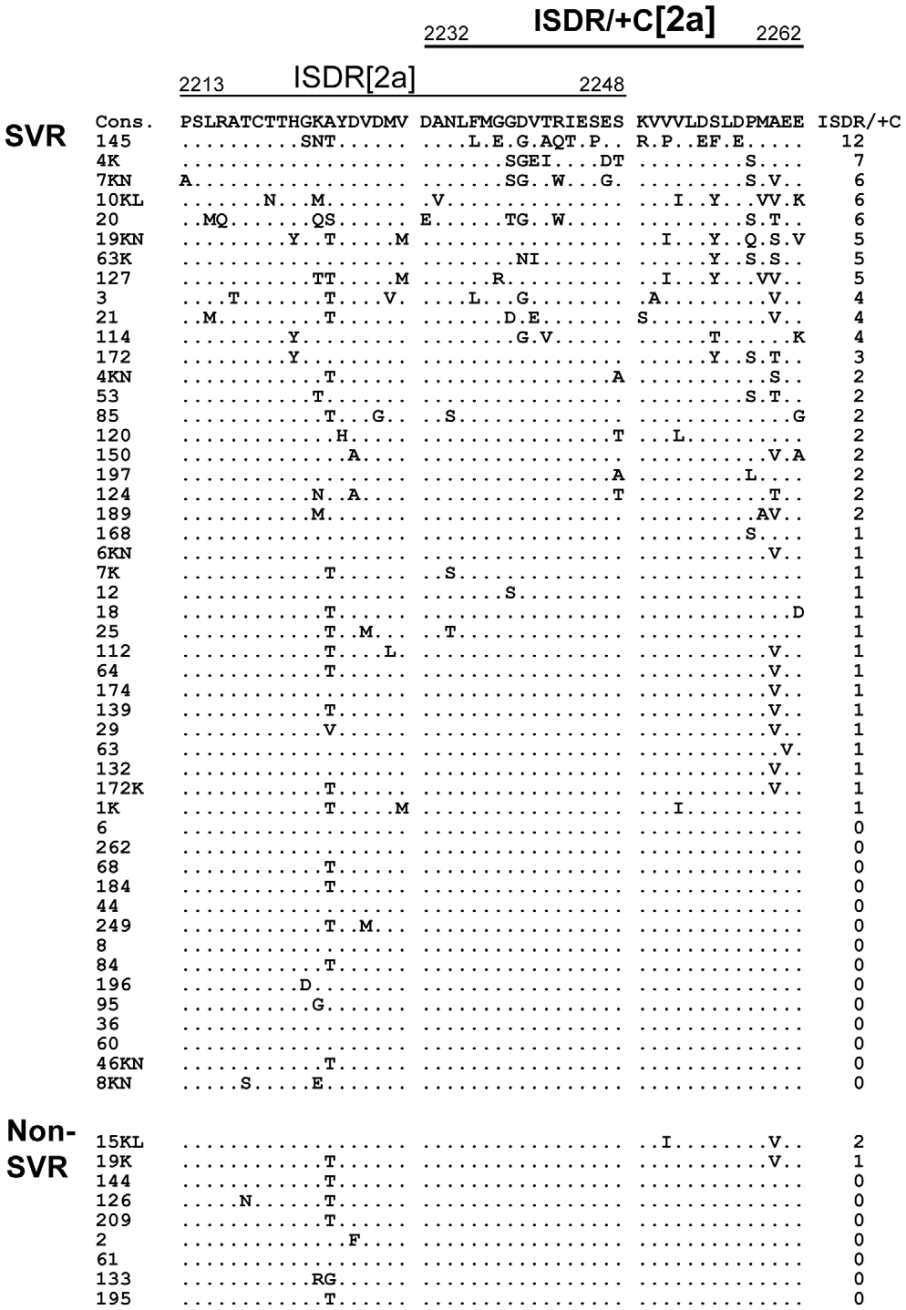
Sequence alignment of ISDR/+C[2a]. Sequences of ISDR/+C[2a] (part of interferon sensitivity determining-region plus its carboxy-flanking region of HCV-2a) obtained from SVR and non-SVR patients are aligned. The consensus sequence (Cons) is shown on the top. The numbers along the sequence indicate the aa positions. Dots indicate residues identical to those of the Cons sequence. The numbers of the mutations in ISDR/+C[2a] are shown on the right.

To investigate the impact of NS5A heterogeneity on the clinical outcome of PEG-IFN/RBV therapy, we first performed a sliding window analysis with a window size of 20 residues over the full-length NS5A sequences obtained from 23 RVR and 7 non-RVR patients infected with HCV-2a along with the consensus sequence, as described previously [8]. This analysis revealed that the number of aa mutations differed significantly between RVR and non-RVR isolates in two regions within the C-terminal half of NS5A (data not shown). The more C-terminally located one exactly matched the region that corresponded to IRRDR of HCV-1b, ranging from aa 2332 to 2387, thus being referred to as IRRDR[2a] (see Figure 1). The other region composed of a part of ISDR plus its carboxy-flanking region, ranging from aa 2232 to 2262, thus being referred to as ISDR/+C[2a] (see Figure 2). It was confirmed that the average numbers of aa mutations in IRRDR[2a] and ISDR/+C[2a] were each significantly larger in isolates from RVR than those from non-RVR patients (Table 3). More importantly, the average numbers of aa mutations in IRRDR[2a] and ISDR/+C[2a] were each significantly larger in SVR than in non-SVR. Sequences of IRRDR[2a] and ISDR/+C[2a] obtained from SVR and non-SVR patients and the number of mutations of each isolate are shown in Figures 1 and 2.

**Table 3:** Average numbers of aa mutations within IRRDR[2a], ISDR/+C[2a] and IRRDR/N[2b] of HCV NS5A obtained form pre-treated sera of HCV-2a and -2b-infected patients with SVR, non-SVR, RVR and non-RVR.

NS5A region	No. of mutations			No. of mutations		
	SVR	Non-SVR	*P* value	RVR	Non-RVR	*P* value
IRRDR[2a] (aa 2332–2387)	6.4±3.4*	3.3±2.1	**0.01**	6.8±3.3	3.3±1.9	0.0003
ISDR/+C[2a] (aa 2232–2262)	2.0±2.4	0.3±0.7	**0.047**	2.1±2.5	0.6±0.7	0.025
IRRDR/N[2b] (aa 2332–2357)	1.8±1.5	1.4±1.3	0.45	2.0±1.4	1.0±1.2	0.01

*Mean ± S.D.

Abbreviations: SVR, sustained virological response; RVR, rapid virological response; IRRDR[2a], interferon/ribavirin resistance-determining region of HCV-2a; ISDR/+C[2a], part of interferon sensitivity determining-region plus its carboxy-flanking region of HCV-2a; IRRDR/N[2b], an N-terminal part of interferon/ribavirin resistance-determining region of HCV-2b.

**Figure 3:**
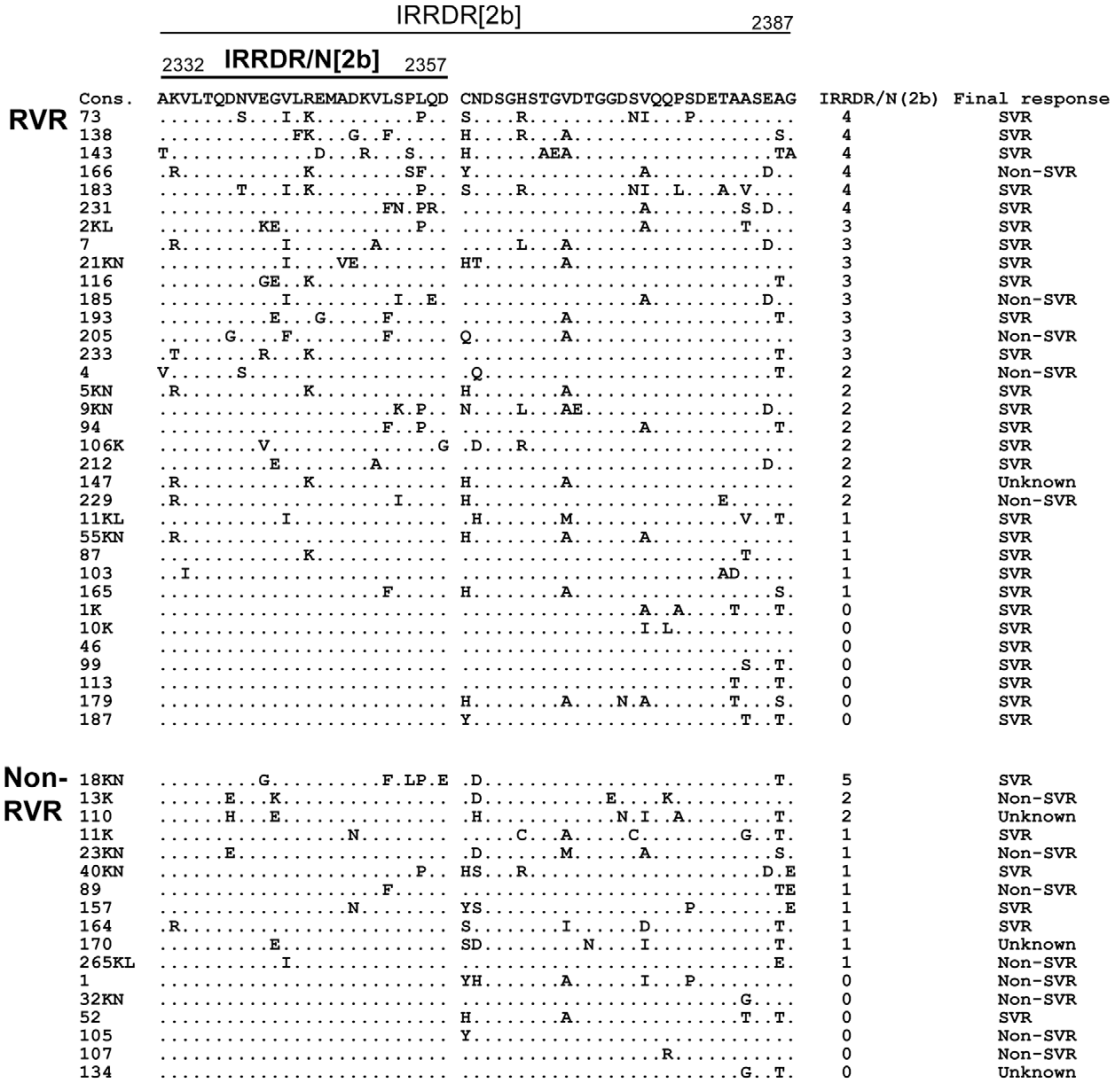
Sequence alignment of NS5A of HCV-2b isolates. Sequences of IRRDR/N[2b] (an N-terminal part of interferon/ribavirin resistance-determining region of HCV-2b) obtained from RVR and non-RVR patients are aligned. The consensus sequence (Cons) is shown on the top. The numbers along the sequence indicate the aa positions. Dots indicate residues identical to those of the Cons sequence. The numbers of the mutations in IRRDR/N[2b] and the final treatment outcome of each patient are shown on the right.

Likewise, a sliding window analysis on HCV-2b isolates (16 RVR and 6 non-RVR) identified an N-terminal part of IRRDR (aa 2332 to 2357), referred to as IRRDR/N[2b], that showed a significant difference in the number of aa mutations between RVR and non-RVR (data not shown). The average numbers of aa mutations in IRRDR/N[2b] were significantly larger in RVR than in non-RVR (Table 3). However, they did not differ significantly between SVR and non-SVR. Sequences of IRRDR[2b]/N obtained from RVR and non-RVR patients are shown in Figure 3.

### Correlation between NS5A Sequence Heterogeneity and SVR or RVR in HCV-2a and HCV-2b infections

The receiver operating characteristic analysis identified the optimal thresholds of the numbers of aa mutations in IRRDR[2a] and ISDR/+C[2a] for the prediction of RVR and SVR in HCV-2a infection; four and one for IRRDR[2a] and ISDR/+C[2a], respectively (data not shown). Accordingly, we found that 86% (42/49) of SVR patients, and only 22% (2/9) of non-SVR, were infected with HCV-2a isolates having IRRDR with 4 or more mutations (IRRDR[2a]≥4) (Table 4). On the other hand, 14% (7/49) of SVR, and 78% (7/9) of non-SVR patients, were infected with isolates having IRRDR with 3 or less mutations (IRRDR[2a]≤3). These results suggested that IRRDR[2a]≥4 was significantly associated with SVR (*P* = 0.0003). Similarly, 93% (42/46) of RVR patients, and only 33% (5/15) of non-RVR, were infected with HCV-2a isolates of IRRDR[2a]≥4 while 7% (4/46) of RVR patients, and 67% (10/15) of non-RVR, were infected with HCV-2a isolates of IRRDR[2a]≤3, with the results suggesting that IRRDR[2a]≥4 was significantly associated with RVR as well (*P*<0.0001).

**Table 4:** Correlation between NS5A sequence heterogeneity and SVR or RVR in HCV-2a and HCV-2b infections.

Factor	SVR	Non-SVR	*P* value	RVR	Non-RVR	*P* value
IRRDR[2a]≥4	42/49* (86%)	2/9 (22%)	**0.0003**	42/46 (93%)	5/15 (33%)	<0.0001
IRRDR[2a]≤3	7/49 (14%)	7/9 (78%)		4/46 (7%)	10/15 (67%)	
ISDR/+C[2a]≥1	35/49 (71%)	2/9 (22%)	**0.008**	32/46 (70%)	7/15 (47%)	0.1
ISDR/+C[2a] = 0	14/49 (29%)	7/9 (78%)		14/46 (30%)	8/15 (53%)	
IRRDR/N[2b]≥2	17/34 (50%)	6/13 (46%)	1.0	22/34 (65%)	3/17 (18%)	0.0025
IRRDR/N[2b]≤1	17/34 (50%)	7/13 (54%)		12/34 (35%)	14/17 (82%)	

*No. of isolates with a given factor/total no. of SVR or RVR.

Abbreviations: SVR, sustained virological response; RVR, rapid virological response; IRRDR[2a], interferon/ribavirin resistance-determining region of HCV-2a; ISDR/+C[2a], part of interferon sensitivity determining-region plus its carboxy-flanking region of HCV-2a; IRRDR/N[2b], an N-terminal part of interferon/ribavirin resistance-determining region of HCV-2b.

As for ISDR/+C[2a] heterogeneity, 71% (35/49) of SVR, and 22% (2/9) of the non-SVR patients, were infected with HCV-2a isolates with ISDR/+C having one or more mutation (ISDR/+C[2a]≥1) (Table 4). On the other hand, 29% (14/49) of SVR patients, and 78% (7/9) of the non-SVR, were infected with isolates with ISDR/+C without mutation (ISDR/+C[2a] = 0). Thus, ISDR/+C[2a]≥1 was significantly associated with SVR (*P* = 0.008).

As for HCV-2b infection, the receiver operating characteristic analysis identified “two” as the optimal threshold of the number of mutations in IRRDR/N[2b] by which to predict RVR (data not shown). Accordingly, we found that 65% (22/34) of RVR, and 18% (3/17) of non-RVR patients, were infected with HCV-2b isolates of IRRDR/N[2b]≥2 (Table 4). On the other hand, 35% (12/34) of RVR, and 82% (14/17) of the non-RVR patients, were infected with IRRDR/N[2b]≤1. These results suggested that IRRDR/N[2b]≥2 was significantly associated with RVR (*P* = 0.0025). However, no correlation, or even no tendency toward significant correlation, was observed between IRRDR/N[2b]≥2 and SVR in HCV-2b infection.

### Correlation between NS5A Sequence Heterogeneity and Viremia Titers in the Serum of patients infected with HCV-2a and HCV-2b before PEG-IFN/RBV Therapy

**Figure 4:**
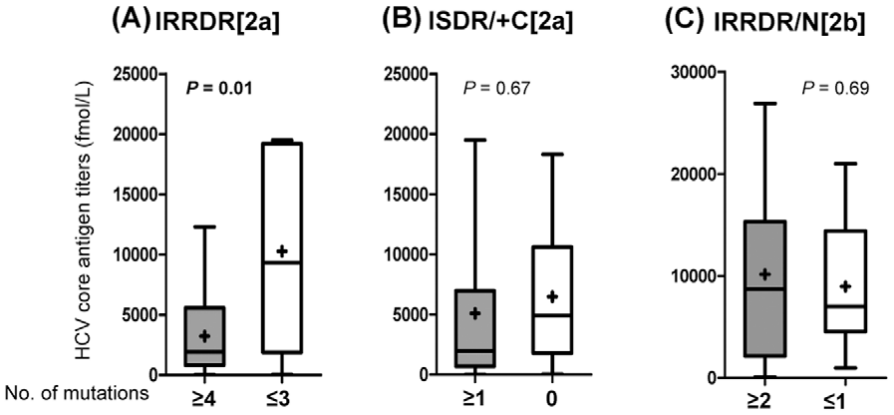
Correlation between NS5A sequence heterogeneity and pretreatment serum HCV core antigen titers in HCV-2a and HCV-2b infections. Pretreatment serum HCV core antigen titers of patients classified on the basis of the number of mutations in IRRDR[2a] (interferon/ribavirin resistance-determining region of HCV-2a) (≥4 vs. ≤3) (A), ISDR/+C[2a] (part of interferon sensitivity determining-region plus its carboxy-flanking region of HCV-2a) (≥1 vs.  = 0) (B) and IRRDR/N[2b] (≥2 vs. ≤1) (an N-terminal part of interferon/ribavirin resistance-determining region of HCV-2b) (C) are depicted. Maximum and minimum values are indicated by the upper and lower bars, respectively. Distribution ranges are displayed as boxes. Mean and median values are also indicated inside the boxes as+and horizontal bars, respectively.

Next, we examined the impact of IRRDR sequence heterogeneity on HCV titers in the serum before the initiation of the treatment. As shown in Figure 4A, patients infected with IRRDR[2a]≥4 had significantly lower pretreatment serum HCV core antigen titers than those infected with IRRDR[2a]≤3. On the other hand, there was no significant difference in HCV viremia titers between ISDR/+C[2a]≥1 and ISDR/+C[2a] = 0 (Figure 4B). Also, in HCV-2b infection, there was no significant difference in pretreatment HCV viremia titers between IRRDR/N[2b]≥2 and IRRDR/N[2b]≤1 (Figure 4C).

### Correlation between Core Protein Sequence Heterogeneity and RVR or SVR

A close correlation between core protein sequence patterns and treatment outcome has been proposed in HCV-1b infection [12], [13]. To examine this hypothesis in HCV-2a and -2b infections, core regions of the virus genome were amplified from the pretreated sera, and the aa sequences deduced and aligned with the prototype sequences (HCV-J6 [18] and HCV-J8 [19]). The residues at positions 70 and 91, which were reported to be associated with the treatment outcome in HCV-1b infection [13], were both well conserved among HCV-2a and -2b isolates and, therefore, no correlation with treatment outcome was expected for these residues (Figures S1 and S2). In this connection, the residues at positions 48 and 110 of HCV-2a isolates showed certain degrees of variation. However, there was no significant correlation between the sequence patterns and the treatment outcome.

### Identification of Independent Predictive Factors for SVR and RVR in HCV-2a and HCV-2b infections

In order to identify significant independent predictors of SVR in HCV-2a and HCV-2b infections, univariate and multivariate logistic regression analyses were carried out using all available data of baseline patients' parameters and viral genetic polymorphic factors. Univariate analysis identified 3 factors that were significantly associated with SVR in HCV-2a infection; the heterogeneity of IRRDR[2a] (≥4 vs. ≤3), ISDR/+C[2a] (≥1 vs.  = 0) and patients' age (<55 years) (Table 5). Subsequently, these factors were entered in multivariate regression analysis. The result obtained revealed that the IRRDR[2a] heterogeneity was the only independent predictive factor for SVR in HCV-2a infection (*P* = 0.001). The IRRDR[2a] heterogeneity was also the independent predictive factor for RVR (Table S1).

**Table 5:** Univariate and multivariate analyses for identification of independent predictive factors for SVR in HCV-2a- and -2b-infected patients treated with PEG-IFN/RBV therapy.

Genotype	Variable	Univariate		Multivariate	
		Odds ratio (95% CI)	*P* value	Odds ratio (95% CI)	*P* value
HCV-2a	IRRDR[2a] mutations	21.0 (3.6–122.5)	0.0003	21.0 (3.6–122.5)	**0.001**
	ISDR/+C[2a] mutations	8.8 (1.6–47.4)	0.008		
	Age (<55 years)	9.8 (1.1–84.7)	0.026		
HCV-2b	γ-GTP (<30 IU/L)	26.0 (1.3–504.7)	0.004	6.2 (1.1–36.2)	0.04
	Body weight (<65 kg)	3.8 (1.0–13.9)	0.06		

Abbreviations: SVR, sustained virological response; IRRDR[2a], interferon/ribavirin resistance-determining region of HCV-2a; ISDR/+C[2a], part of interferon sensitivity determining-region plus its carboxy-flanking region of HCV-2a; γ-GTP, gamma glutamyl transpeptidase.

As for HCV-2b infection, univariate analysis identified two host factors that were significantly, or almost significantly, associated with SVR; γ-GTP levels (<30 IU/L) and body weight (<65 kg) (Table 5). No viral factor was identified in this analysis. In subsequent multivariate analysis, γ-GTP levels was identified as an independent predictive factor for SVR in HCV-2b infection. In this connection, the heterogeneity of IRRDR/N[2b], a viral factor, was identified to be significantly associated with RVR in HCV-2b infection (Table S1).

## Discussion

The clinical outcome of PEG-IFN/RBV therapy for HCV infection is influenced by a number of host and viral factors [20]. It has recently been reported that host genetic polymorphisms near or within the IL28B gene on the chromosome 19 show a critical impact on the treatment outcome of patients infected with HCV-1a and -1b [21]–[23]. Also, HCV genetic polymorphisms have been known to contribute to differences in the treatment outcome, as demonstrated by the observations that SVR rates for patients infected with HCV genotypes 2 and 3 are higher than those for patients infected with HCV genotype 1 [2], [6]. Moreover, polymorphisms of NS5A and core regions of a given HCV genotype, in particular HCV-1b, have been linked to the difference in SVR rates [7], [8], [11]–[13], [17]. It should be noted that the significant link between polymorphisms of NS5A and core regions of HCV-1b and treatment outcome was inferred mostly from studies carried out on patients in Asian countries, in particular Japan, and that somewhat controversial results were obtained from studies carried out on patients infected with HCV-1a or -1b in non-Asian countries [24]–[31]. However, we would like to point out that most of these publications focused mainly on ISDR and core mutations, but not on IRRDR. In addition, the impact of viral genetic variation on treatment outcome in non-HCV-1 infection, either in Asian or non-Asian countries, is still unclear.

In our previous study, we identified IRRDR in NS5A of HCV-1b as a significant determinant for PEG-IFN/RBV treatment outcome; EVR and, more importantly, SVR [11], [12]. Consistent with the previous observation, we have demonstrated in the present study that sequence heterogeneity within IRRDR is closely correlated with the treatment responses in HCV-2a and -2b infections. In HCV-2a infection, IRRDR[2a]≥4 was closely associated with RVR (Table S1) and SVR (Table 5). In HCV-2b infection, the sequence heterogeneity within an N-terminal part of IRRDR (IRRDR/N[2b]) was significantly associated with RVR (Table S1). Furthermore, both IRRDR[2a]≥4 and ISDR/+C[2a]≥1 showed remarkable positive predictive values (95%) for SVR prediction (Table S2), suggesting the clinical usefulness of these markers to encourage those patients to receive PEG-IFN/RBV treatment. On the other hand, their negative predictive values for non-SVR were rather low (50% and 33%). This suggests the possible involvement of another factor(s) that determines non-SVR and may limit the clinical usefulness of these markers to accurately predict non-SVR.

The present results were dependent upon the small number of non-SVR patients due to the high response rates of HCV-2a and -2b. In spite of this, the parallels between the RVR/non-RVR and the SVR/non-SVR analyses, especially in HCV-2a infection, support the possibility that the sequences presented in this study are truly representative of the viruses in general circulation.

**Figure 5:**
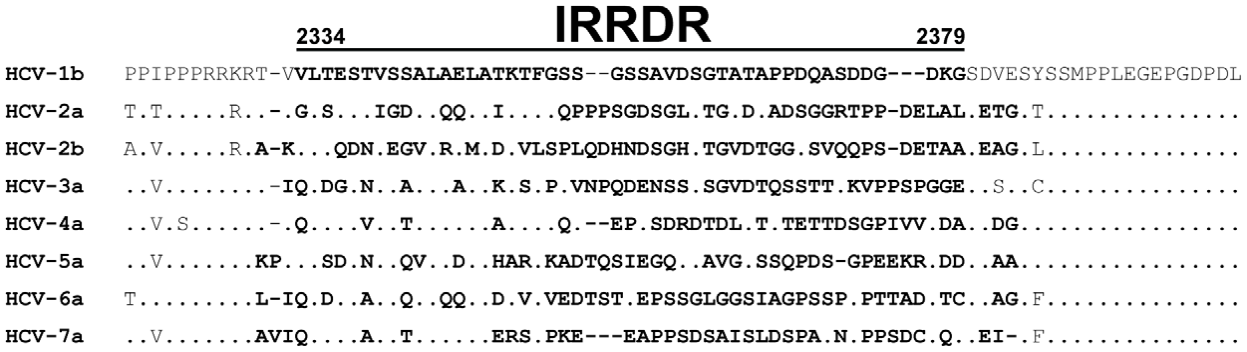
Sequence alignment of IRRDR (interferon/ribavirin resistance-determining region) and its upstream and downstream sequences of different HCV genotypes. The residues in the region that corresponds to IRRDR of HCV-1b [11] are written in boldface letters. Dots indicate residues identical to the HCV-1b sequence. References of aligned sequences are: HCV-1b, El-Shamy et al. [11]; HCV-2a and -2b, Murakami et al. [8]; HCV-3a, X76918; HCV-4a, Y11604; HCV-5a, AF064490; HCV-6a, D84262; HCV-7a, EF108306.

The clinical correlation between IRRDR sequence heterogeneity and virological responses of IFN-based therapy in HCV infection can be linked to a recent experimental observation by Tsai et al. [32] that an HCV subgenomic RNA replicon containing NS5A of HCV-1b exerted more profound inhibitory effects on IFN activities than the original HCV-2a replicon, and that domain swapping between NS5A sequences of HCV-1b and -2a in the V3 and/or a C-terminal region including IRRDR resulted in a transfer of their anti-IFN activities. Also, it is worthy to note that IRRDR is among the most variable sequences across the different genotypes and subtypes of HCV [33] whereas its upstream and downstream sequences show a higher degree of sequence conservation (Figure 5). This may suggest that whereas the upstream and downstream sequences have a conserved function(s) across all the HCV genotypes, IRRDR sequences have a genotype-dependent or even a strain-dependent function(s). Indeed, the upstream sequences, especially a Pro-rich motif, play key roles in multiple stages of viral replication [34] while the downstream sequence in viral particle assembly and production [35]. Therefore, the sequence heterogeneity of IRRDR and its significant correlation with IFN-responsiveness imply the possibility that IRRDR is involved, at least partly, in the viral strategy to evade IFN-mediated antiviral host defense mechanisms. Its possible molecular mechanism, however, is yet to be elucidated. The IRRDR sequence heterogeneity also suggests genetic flexibility of this region and, indeed, the C-terminal portion of NS5A was shown to tolerate sequence insertions and deletions [36]. This flexibility might play an important role in modulating the interaction with various host systems, including IFN-induced antiviral machineries. It is also possible that the genetic flexibility of IRRDR is accompanied by compensatory changes elsewhere in the viral genome and that these compensatory changes affect overall viral fitness and responses to IFN-based therapy [37].

The relapse rate was higher in HCV-2b infection than in HCV-2a (Table 1). It should be noted that while the sequence heterogeneity within IRRDR[2a] was significantly correlated with both RVR and SVR in HCV-2a infection, IRRDR/N[2b] was correlated only with RVR in HCV-2b infection. These observations might be linked to an intrinsic difference in IFN- and/or RBV-sensitivity between HCV-2a and -2b isolates [8], [38]. We assume that HCV-2b is considered between HCV-1b and HCV-2a in terms of resistance to PEG-IFN/RBV treatment and that an extended treatment for a total of 36∼48 weeks would be needed to prevent relapse in HCV-2b infection, especially for patients who have risk factors that do not fit the SVR or RVR prediction criteria (Table 5 and Table S1).

A mutation at position 70 of the core protein of HCV-1b has been reported to be correlated with PEG-IFN/RBV treatment outcome [12], [13]. In the present study, however, we found no significant correlation between core protein polymorphism and treatment outcome in HCV-2a or -2b infections. The residue at position 70 of the core protein of HCV-2a and -2b isolates was Arg, which is known to be associated with SVR in HCV-1b infection [12], [13], and was well conserved in all the isolates tested in the present study (Figures S1 and S2). The observed sequence conservation at position 70 might be the reason for the lack of significant correlation between core protein polymorphism and treatment outcome in HCV-2a or -2b infections. On the other hand, Thr at position 110 of the core protein of HCV-2a has recently been reported to be significantly associated with SVR [10]. In the present study, Thr at position 110 was found in 35% (14/40) and 14% (1/6) of SVR and non-SVR cases, respectively (Figure S1). Similarly, Thr at position 48 was found in 35% (14/40) of SVR cases, but not in non-SVR cases (0/6). The observed differences between SVR and non-SVR, however, were not statistically significant due possibly to the small number of samples tested. A larger-scale study would be needed to determine the possible importance of those residues.

We preliminarily analyzed a host genetic factor, the single nucleotide polymorphism (SNP) at rs8099917 near the IL28B gene [21]–[23], of a portion of the patients examined in the present study. The result showed that the minor genotypes (T/G and G/G) were found in 5.1% (2/39) and 15.4% (2/13) of RVR and non-RVR patients, respectively, and 2.8% (1/36) and 20.0% (2/10) of SVR and non-SVR patients, respectively (Kim et al., unpublished observation). Although the differences were not statistically significant due probably to the small number of the patients tested, the minor genotypes showed a trend toward being associated with non-SVR, and with non-RVR to a lesser extent, in HCV-2a and -2b infections, as has been reported for HCV-1a and -1b infections [21]–[23]. The impact of the IL28B SNP, however, appeared to be weaker in HCV-2a and -2b infections than that seen in HCV-1a and -1b infections, and also weaker than that of the most powerful viral factor, IRRDR[2a]≥4, in HCV-2a infection. In this context, we found that, of the four patients with the minor IL28B genotypes, two patients (nos. 2 and 105), who underwent unfavorable treatment response (non-RVR and non-SVR), were infected with HCV isolates of IRRDR[2a]≤3 or IRRDR/N[2b]≤1 while the other two patients (no. 63 and 106), who achieved favorable treatment response (SVR and/or RVR), were infected with HCV isolates of IRRDR[2a]≥4. This might imply the possibility that, in HCV-2 infection, the combination of the minor IL28B genotypes and a low degree of IRRDR sequence heterogeneity has a strong power to predict unfavorable treatment responses whereas a high degree of IRRDR sequence heterogeneity has a dominant predictive power for favorable treatment responses regardless the IL28B genotype. Analysis in a large-scale multicenter study is needed to clarify this issue.

In conclusion, our data suggest that the sequence heterogeneity of NS5A, i.e., IRRDR[2a]≥4, and ISDR/+C[2a]≥1 to a lesser extent, would be a useful predictive marker for SVR in HCV-2a infection. Also, IRRDR/N[2b]≥2 is significantly associated with RVR in HCV-2b infection. These results further emphasize the importance of NS5A, a viral factor, in determining the responsiveness to PEG-IFN/RBV therapy.

## Supporting Information

Figure S1 **Sequence alignment of the core protein of HCV-2a isolates.** Core protein sequences (aa 1 to 120) of HCV-2a obtained from SVR and non-SVR patients are aligned. Prototype sequence of HCV-J6 [18] is shown on the top. The numbers along the sequence indicate the aa positions. Dots indicate residues identical to those of the prototype sequence.
(TIF)

Figure S2 **Sequence alignment of the core protein of HCV-2b isolates.** Core protein sequences (aa 1 to 120) of HCV-2b obtained from SVR and non-SVR patients are aligned. Prototype sequence of HCV-J8 [19] is shown on the top. The numbers along the sequence indicate the aa positions. Dots indicate residues identical to those of the prototype sequence.
(TIF)

Table S1 **Univariate and multivariate analyses for identification of independent predictive factors for RVR in HCV-2a- and -2b-infected patients treated with PEG-IFN/RBV therapy.**
(DOC)

Table S2 **Positive and negative predictive values (PPV and NPV) of NS5A polymorphic factors for SVR prediction.**
(DOC)
