# Supplementary figures and images for: Sequence Heterogeneity in NS5A of Hepatitis C Virus Genotypes 2a and 2b and Clinical Outcome of Pegylated-Interferon/Ribavirin Therapy
Source: PLoS One. 2012 Feb 2;7(2):e30513. doi: 10.1371/journal.pone.0030513 (PMC3271109; doi:10.1371/journal.pone.0030513)

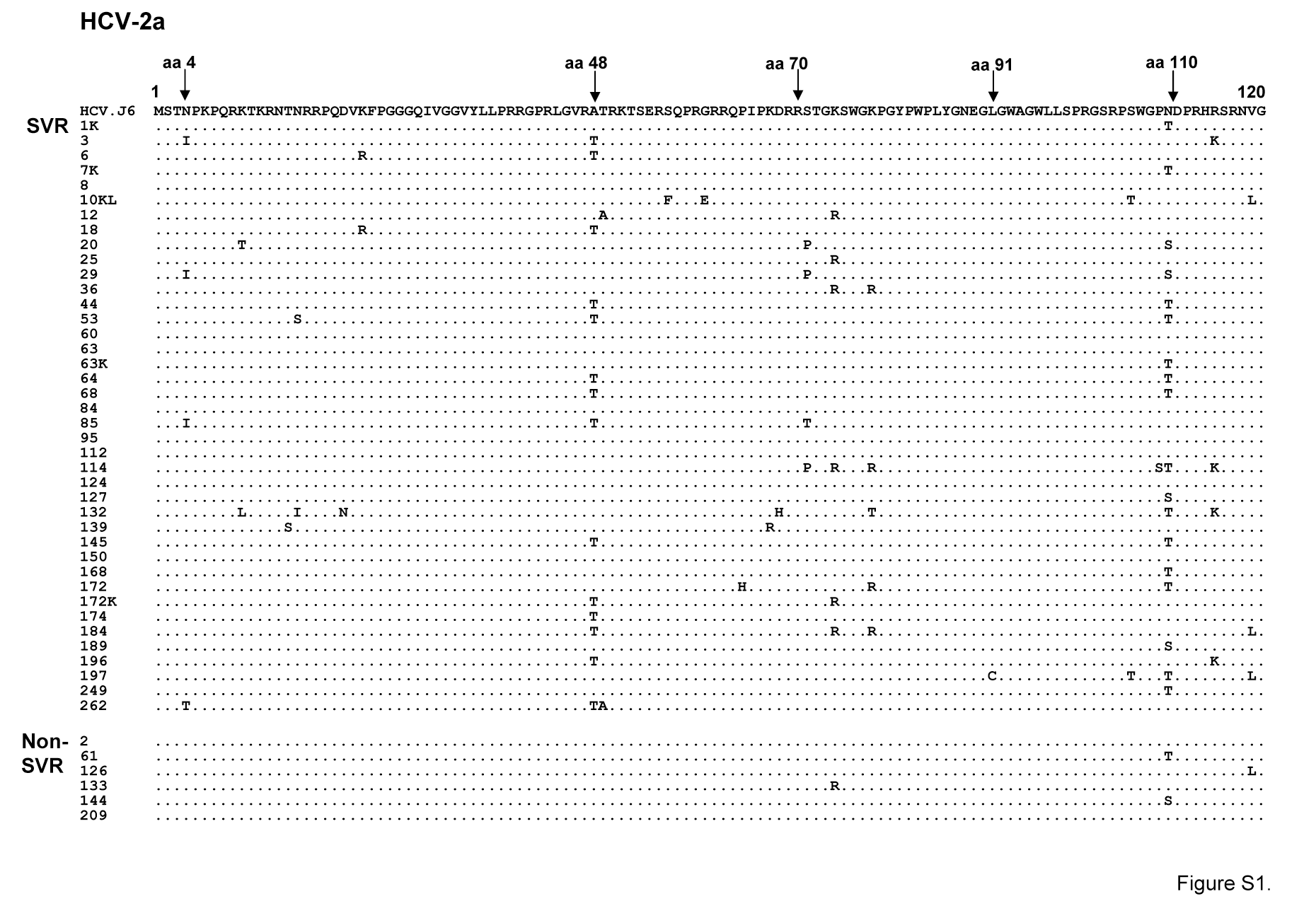

Supplement: Figure S1 — Sequence alignment of the core protein of HCV-2a isolates. Core protein sequences (aa 1 to 120) of HCV-2a obtained from SVR and non-SVR patients are aligned. Prototype sequence of HCV-J6 [18] is shown on the top. The numbers along the sequence indicate the aa positions. Dots indicate residues identical to those of the prototype sequence. (TIF) [file pone.0030513.s001.tif]

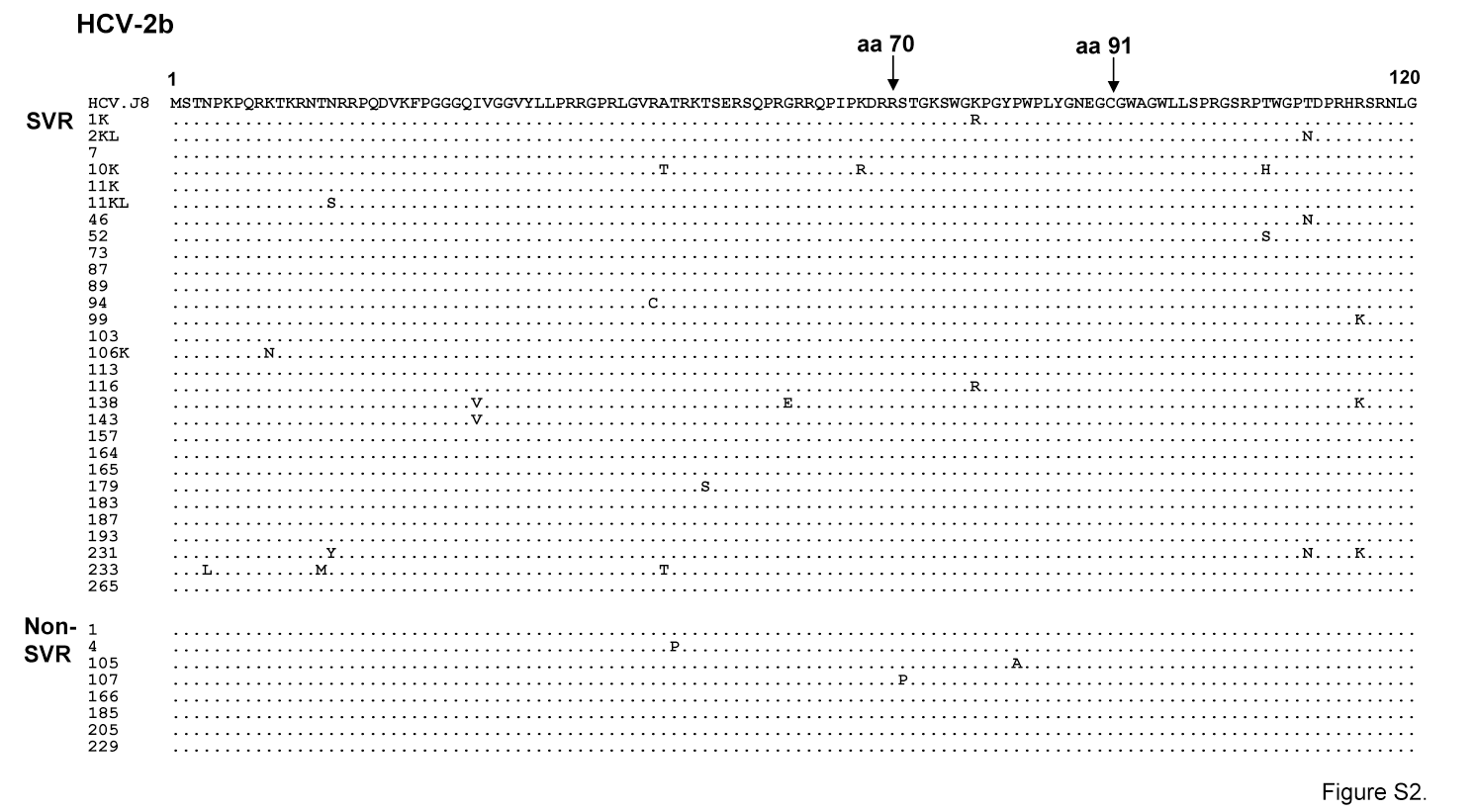

Supplement: Figure S2 — Sequence alignment of the core protein of HCV-2b isolates. Core protein sequences (aa 1 to 120) of HCV-2b obtained from SVR and non-SVR patients are aligned. Prototype sequence of HCV-J8 [19] is shown on the top. The numbers along the sequence indicate the aa positions. Dots indicate residues identical to those of the prototype sequence. (TIF) [file pone.0030513.s002.tif]
