# Supplementary material for: Sequence Heterogeneity in NS5A of Hepatitis C Virus Genotypes 2a and 2b and Clinical Outcome of Pegylated-Interferon/Ribavirin Therapy
Source: PLoS One. 2012 Feb 2;7(2):e30513. doi: 10.1371/journal.pone.0030513 (PMC3271109; doi:10.1371/journal.pone.0030513)
Supplement: Table S1 — Univariate and multivariate analyses for identification of independent predictive factors for RVR in HCV-2a- and -2b-infected patients treated with PEG-IFN/RBV therapy. (DOC) [file pone.0030513.s003.doc]

**Table S1.** Univariate and multivariate analyses for identification of independent predictive factors for RVR in HCV-2a- and -2b-infected patients treated with PEG-IFN/RBV therapy.

| Genotype | Variable | Univariate | | Multivariate | |
| --- | --- | --- | --- | --- | --- |
|  |  | Odds ratio (95% CI) | *P* value | Odds ratio (95% CI) | *P* value |
| HCV-2a | IRRDR[2a] mutations | 21.0 (4.8 – 92.8) | <0.0001 | 60.0 (4.6 – 782.4) | 0.002 |
|  | Age (<55 years) | 3.9 (1.1 – 14.2) | 0.04 |  |  |
|  | Sex | 8.5 (1.7 – 41.8) | 0.006 |  |  |
|  | ALT (> 30 IU/L) | 0.1 (0.03 – 0.4) | 0.0008 |  |  |
|  | HCV core antigen (<7,000 fmol/L) | 11.3 (1.6 – 80.3) | 0.016 |  |  |
| HCV-2b | IRRDR/N[2b] mutations | 8.6 (2.0 – 35.8) | 0.003 | 6.1 (1.3 – 27.1) | 0.018 |
|  | Age (<40 years) | 17.1 (0.9 – 311.0) | 0.01 |  |  |

Abbreviations: RVR, rapid virological response; IRRDR[2a], interferon/ribavirin resistance-determining region of HCV-2a; ISDR/+C[2a], part of interferon sensitivity determining-region plus its carboxy-flanking region of HCV-2a; ALT, alanine aminotransferase; IRRDR/N[2b], an N-terminal part of interferon/ribavirin resistance-determining region of HCV-2b.
