# Supplementary material for: Sequence Heterogeneity in NS5A of Hepatitis C Virus Genotypes 2a and 2b and Clinical Outcome of Pegylated-Interferon/Ribavirin Therapy
Source: PLoS One. 2012 Feb 2;7(2):e30513. doi: 10.1371/journal.pone.0030513 (PMC3271109; doi:10.1371/journal.pone.0030513)
Supplement: Table S2 — Positive and negative predictive values (PPV and NPV) of NS5A polymorphic factors for SVR prediction. (DOC) [file pone.0030513.s004.doc]

**Table S2.** PPV and NPV of NS5A polymorphic factors for SVR prediction.

| Predictive marker | PPV% | NPV% |
| --- | --- | --- |
| IRRDR[2a] ≥4 | 95% (42/44)* |  |
| IRRDR[2a] ≤3 |  | 50% (7/14)^†^ |
| ISDR/+C[2a] ≥1 | 95% (35/37) |  |
| ISDR/+C[2a] = 0 |  | 33% (7/21) |
| IRRDR/N[2b] ≥2 | 74% (17/23) |  |
| IRRDR/N[2b] ≤1 |  | 29% (7/24) |

* No. of SVR with HCV isolates of a given marker / total no. of HCV isolates of a given marker.

^†^ No. of Non-SVR with HCV isolates of a given marker / total no. of HCV isolates with a given marker.

Abbreviations: SVR, sustained virological response; IRRDR[2a], interferon/ribavirin resistance-determining region of HCV-2a; ISDR/+C[2a], part of interferon sensitivity determining-region plus its carboxy-flanking region of HCV-2a; IRRDR/N[2b], an N-terminal part of interferon/ribavirin resistance-determining region of HCV-2b; PPV, positive predictive value; NPV, negative predictive value.
